# Morphological and molecular data on acuariid nematodes in European great cormorants (*Phalacrocorax carbo sinensis*) and pygmy cormorants (*Microcarbo pygmaeus*)

**DOI:** 10.1038/s41598-024-64678-1

**Published:** 2024-06-14

**Authors:** Perla Tedesco, Monica Caffara, Nadav Davidovich, Valentina Luci, Alessia Cantori, Maria Letizia Fioravanti, Andrea Gustinelli

**Affiliations:** 1 Department of Veterinary Medical Sciences (DIMEVET), Alma Mater Studiorum University of Bologna, Ozzano Emilia, Italy; 2 Israeli Veterinary Services, Beit Dagan, Israel

**Keywords:** Acuariidae, Piscivorous birds, *Syncuaria squamata*, *Cosmocephalus obvelatus*, Italy, Israel, Parasite biology, Parasite genetics

## Abstract

The family Acuariidae is a speciose group of parasitic nematodes, infecting mostly birds as definitive hosts. This study focused on the characterization of two species of acuariids, collected in two different species of piscivorous birds, the European great cormorant *Phalacrocorax carbo sinensis* from Italy, and the pygmy cormorant *Microcarbo pygmaeus* from Israel. Parasites were analyzed using light and scanning electron microscopy and by amplification and sequencing of the 28S rDNA. The results of morphological and molecular analyses showed that *Ph. carbo sinensis* was infected by the acuariid *Syncuaria squamata* (12 females) and *Cosmocephalus obvelatus* (1 female), whereas *M. pygmaeus* was infected by *C. obvelatus* (2 males, 12 females). The present results provide new data on the distribution of acuariid parasites of piscivorous birds, the first report of Acuariidae in Israel, and the first molecular data on *S. squamata* and *C. obvelatus*, which will be useful in future epidemiological and phylogenetic studies of these widely distributed, but less molecularly studied parasites.

## Introduction

The family Acuariidae Railliet, Henry and Sisoff, 1912 (Chromadorea: Rhabditida) comprises more than 300 species of parasitic nematodes, which are mostly found in aquatic and terrestrial birds^1–3^, except for few genera that are specific parasites of mammals^2,4,5^.

Members of this group have a complex life cycle; in aquatic species, birds are definitive hosts, while different arthropods and fish are intermediate and paratenic hosts, respectively. For some of these aquatic species, details of the life cycle have been demonstrated through either experimental studies or field observations^5,6^. Acuariid larvae have been reported in different groups of arthropod hosts (Wong & Anderson^7^, and references therein), including brine shrimps^8^, crabs^9^, ostracods^10,11^ and dragonfly larvae^5^, nevertheless, for the majority of acuariid species, the identity of intermediate/paratenic hosts is currently unknown. In the bird host, acuariids parasitize the proventriculus, under the koilin of the gizzard and, rarely oesophagus and intestine. Some species can be highly pathogenic and even cause mortality in massive infections^2^.

The morphology of anterior cuticular ornamentations is the most important diagnostic feature for distinguishing genera within Acuariidae in addition to the morphology of deirids and the number of precloacal papillae in adult males^3,12^.

Several species show restricted host and geographic distribution, while others have been reported in a wide range of bird species and appear to have cosmopolitan distribution. However, a number of these records refers to dated literature, with often incomplete morphological descriptions. The increasing use of molecular data in taxonomic studies may help understand diversity patterns among generalist and cosmopolitan acuariid species, although to date few representatives of this large family have been genetically characterized.

The present study was focused on the identification of acuariid parasites from two cormorant species, the European great cormorant *Phalacrocorax carbo sinensis* (Blumenbach, 1798) sampled in Italy, and the pygmy cormorant *Microcarbo pygmaeus* (Pallas, 1773) collected in Israel, providing morphological and morphometric descriptions by light and scanning electron microscopy and sequence data of the 28S rDNA.

## Materials and methods

Nematodes were extracted from the intestine of 1 *Ph. carbo sinensis* found dead (Emilia Romagna, Italy in 2023), and from the gastric mucosa of 3 *Microcarbo pygmaeus* (2 from Mevo Hama and 1 from Kfar Ruppin, Israel in 2021) shot and processed fresh. For the birds from Israel all experimental protocols were approved under permit 2020/42686 from the Israel Nature and Parks Authority; all methods were carried out in accordance with relevant guidelines and regulations; all methods are reported in accordance with ARRIVE guidelines (https://arriveguidelines.org). In both cases the worms were carefully washed in saline and cleaned with a small brush, then preserved in 70% ethanol for downstream analyses. Few specimens from each locality were also preserved in 10% neutral buffered formalin for SEM studies.

The nematodes were observed under a dissection microscope to first evaluate gross morphology and measure the total length (TL), then under a light microscope (Leica Microsystems, Wetzlar, Germany) with the aid of a digital Nikon DS-Fi1 camera and image-acquisition software (Nikon Nis-Elements D3.0). A section of each worm was removed for DNA extraction (central 5 mm, devoided of taxonomically informative features). Anterior and posterior portions were then clarified in Amman’s lactophenol to measure internal structures by light microscope. Species identification was carried out following^1,13,14^.

For SEM, anterior and posterior portions of the worms were dehydrated through a graded ethanol series, subjected to critical point drying, sputter-coated with gold palladium, and observed using a Phenom XL G2 Desktop SEM (Thermo Fisher Scientific, Eindhoven, The Netherlands) operating at 5 kV.

For the molecular analysis, the genomic DNA from 20 specimens (13 from Italy and 7 from Israel) was extracted using PureLink® Genomic DNA Kit (Life Technologies, Carlsbad, California) following the manufacturer’s instructions. The amplification of the D1–D3 variable region of the 28S rDNA was performed with primers U178_f (5′-GCACCCGCTGAAYTTAAG-3′) and L1642_r (5′-CCAGCGCCATCCATTTTCA-3′)^15^. The thermal cycler program (Tpersonal, Biometra) was 40 cycles of 30 s at 94 °C, 30 s at 52 °C and 2 min at 72 °C, preceded by a denaturation step at 94 °C for 2 min and followed by an extended elongation step at 72 °C for 10 min. The PCR products were electrophoresed on 1% agarose gel stained with SYBR® Safe DNA Gel Stain (Thermo Fisher Scientific, Carlsbad, California) in 0.5X TBE. By NucleoSpin Gel and PCR Cleanup (Mackerey-Nagel, Düren, Germany) the amplicons were purified and sequenced with an ABI 3730 DNA analyzer (StarSEQ Mainz, Germany). The DNA trace files were assembled with ContigExpress (VectorNTI Advance 11 software, Invitrogen, Carlsbad, California) and the consensus sequences were compared with previously published data by BLAST tools (https://blast.ncbi.nlm.nih.gov/Blast.cgi). Multiple sequences alignments were constructed using BioEdit 7.2.5 together with some of the sequences reported by Mutafchiev et al.^3^. Pairwise distance, and maximum likelihood (ML) tree (GTR + G, bootstrap of 1,000 replicates) were obtained by MEGA 7.

## Results

A total of 13 acuariid nematodes (all females) were recovered from *Ph. carbo sinensis* and morphologically identified as *Syncuaria squamata* (Linstow, 1883) except one identified as *Cosmocephalus obvelatus* (Creplin, 1825) Seurat, 1919, while 14 acuariids (2 males, 12 females) were found in *M. pygmaeus* (intensity ranging from 1 to 10) and morphologically identified as *C. obvelatus*. Morphometric data of the specimens analyzed are reported in Tables 1 and 2 and are expressed in micrometres (µm), unless otherwise stated.

**Table 1:** Morphometric data from adult male and female *Syncuaria squamata* examined in this study, along with a comparison to previously documented descriptions.

Host	*Ph. carbo sinensis*, (n = 12)	*Ph. auritus*, ()	*Ph. brasilianus*, ()
Locality	Italy	Canada	Brazil
Reference	Present study	Wong et al., 1986^13^	Monteiro et al. 2006^21^
Body length (mm)	17–29	25.5–32	20.6–30
Maximum body width	394–651	650–800	580–760
Cordons length	739–1160	na	1000–1400
Cordons width	46–69	na	55–62
Buccal cavity	334–516	450–550	420–540
Muscular oesophagus	800–950	700–1200	900–1100
Glandular oesophagus	5400–7600	6000–9400	2400–2600
Deirid length	38–73	na	72–85
Deirid width	na	na	55–70
Deirids, distance from anterior end	786–1232	1200–1400	1100–1500
Nerve ring, distance from anterior end	340–611	510–640	470–620
Vulva, distance from posterior end	200–365	260–320	280–410
Tail	80–123	65–115	92–110
Eggs	30–41 × 20–27 (n = 10)	30–35 × 19–23 (n = 20)	28–35 × 18–25

na = not available.

**Table 2:** Morphometric data from adult male and female *Cosmocephalus obvelatus* examined in this study, along with a comparison to previously documented descriptions.

Host	*Microcarbo pygmaeus*,	Several bird’s species	*Larus delawarensis*	*Eudyptes cristatus*	*Spheniscus magellanicus*	*Larus argentatus*	*Larus canus*
Locality	Israel	Europe	Canada	Chile	Argentina	Bulgaria	Korea
Reference	Present study	Cram, 1927^36^	Anderson & Wong, 1981^43^	Azuma et al. 1988^40^	Diaz et al. 2001^31^	Mutafchiev et al. 2010^14^	Kim et al. 2015^42^
**Male**	n = 2	n = na	n = 10	n = 8	n = 9	n = 10	n = 2
Body length (mm)	9–10.4	5.7–12.2	9.9–14.3	9.6–13.0	8.08–10.4	9.8–11.2	9.1–9.3
Maximum body width	298–306	240–255	200–350	240–300	195–390	255–286	224–246
Cordons length	327–347	na	na	380–520	na	322–376	399–407
Cordons width	29–33	na	na	na	na	27–32	na
Vestibule/buccal cavity/pharynx	344–372	na	380–510	440–500	360–481	340–411	na
Muscular oesophagus	914–1096	na	1000–1300	800–1080	680–1060	848–940	na
Glandular oesophagus	4128–4426	na	3600–4300	2760–4080	2520–3990	2927–3640	na
Deirids, distance from anterior end	416–427	430	na	440–600	369–671	na	na
Nerve ring, distance from anterior end	403–419	na	na	460–580	390–552	na	na
Left spicule	498–527	420–540	590–700	560–640	474–575	487–548	na
Right spicule	123–137	130–155	180–220	160–180	127–212	142–167	na
Tail	247–296	420	400–500	380–440	285–373	290–369	na
**Female**	n = 8	n = na	n = 10	n = 10	n = 10	n = 9	n = 3
Body length (mm)	9.7–15.9	9.7–20	15.8–22.3	11.7–22.8	13.5–22.12	14.8–18.2	15.5–15.9
Maximum body width	299–383	300–380	320–500	280–480	296–627	402–456	302–342
Cordons length	447–495	na	na	420–800	na	452–532	501–548
Cordons width	53–72	na	na	na	na	54–77	na
Buccal cavity/pharynx	407–467	na	570–730	480–760	525–637	456–519	na
Muscular oesophagus	967–1198	na	1200–1500	800–1560	720–1200	1055–1284	na
Glandular oesophagus	3895–4747	na	4100–5100	2320–5240	3160–4980	3922–4375	na
Deirids from anterior end	501–579	490	na	450–900	611–793	na	na
Nerve ring from anterior end	459–552	na	na	440–840	585–780	na	na
Vulva, distance from posterior end	Na	na	na	4300–13,600	na	na	na
Vulva, distance from anterior end	Na	5500	7400–10,400	na	6207–9230	7800–9800	na
Tail	217–246	230	220–380	200–300	182–373	197–291	na
Eggs	30–36 × 17–20 (n = 10)	36 × 20	na	31–37 × 18–22	33–40 × 18–21	35–39 × 20–22 (n = 20)	36–40 × 19–21 (n = 20)

na = not available.

### Morphological description

#### *Syncuaria squamata* (von Linstow, 1883) Wong, Anderson & Bartlett, 1986

**Figure 1:**
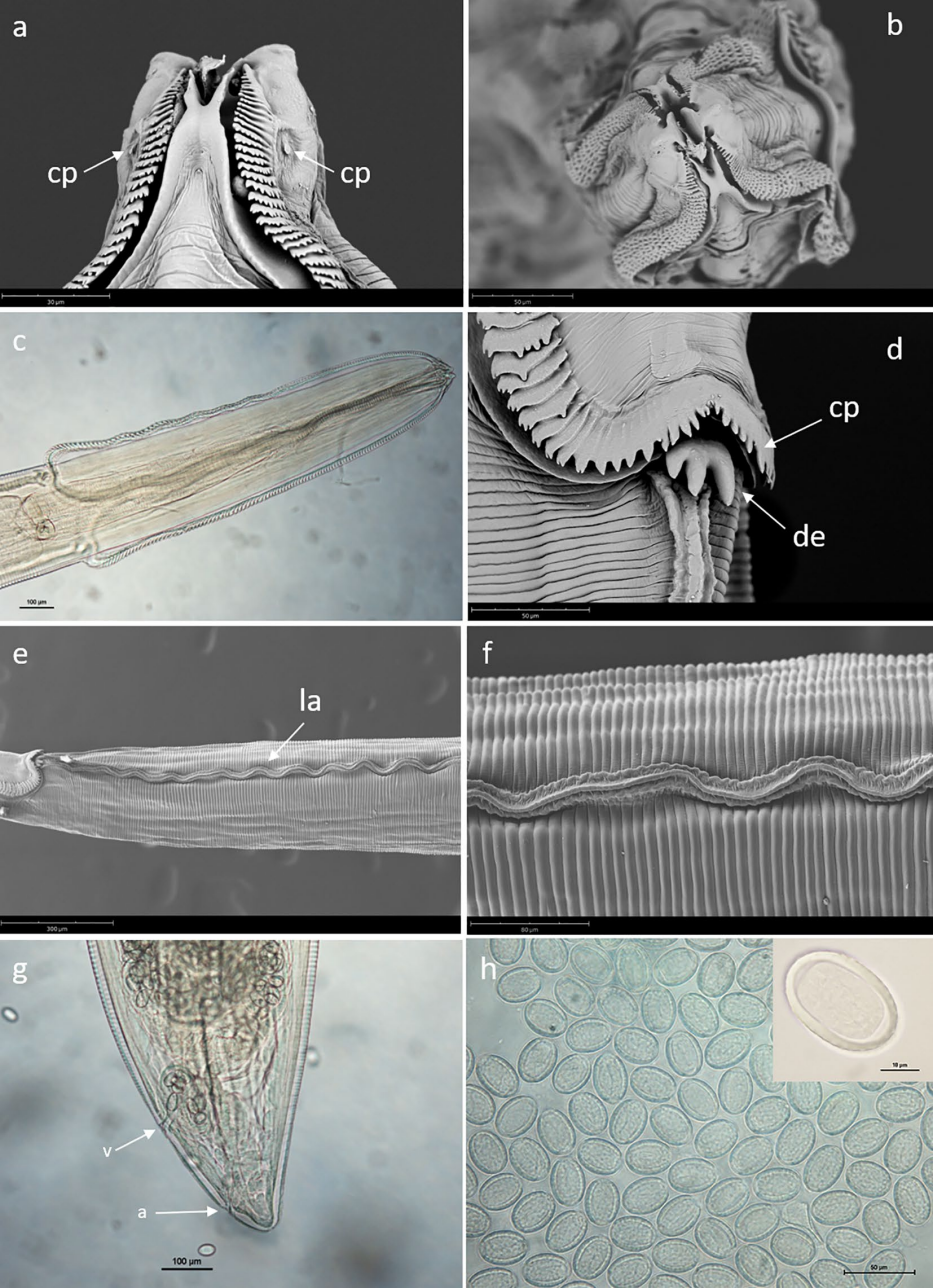
Female specimen of *Syncuaria squamata* from *Phalacrocorax carbo sinensis*, light microscopy and SEM micrographs. (**a**) Anterior end, sublateral view; cephalic papillae (cp) (scale bar = 30 µm). (**b**) Apical view of anterior end showing slit-like oral opening surrounded by two pseudolabia and two bifid interlabia (scale bar = 50 µm). (**c**) Dorsoventral view of anterior end (scale bar = 100 µm). (**d**) Detail of cuticular plates (cp) of cordons and tricuspid deirid (de) (scale bar = 50 µm). (**e**) Lateral alae (la) in anterior part of body (scale bar = 300 µm). (**f**) Detail of lateral alae immediately posterior to cordons (scale bar = 80 µm). (**g**) Posterior end showing the position of vulva (v) and anus (a) (scale bar = 100 µm). (**h**) Eggs (scale bar = 50 µm) (inset: detail of one egg at higher magnification. Scale bar = 10 µm).

Adult female morphology: oral opening slit-like, surrounded by two pseudolabia, each with a single amphid and a pair of papillae, and two bifid interlabia (Fig. 1a,b). Narrow cordons, originating dorsally and ventrally to pseudolabia, anastomising laterally, formed by serrated, crescent-shaped, cuticular plates (Fig. 1c–d). Deirids tricuspid (Fig. 1d). Nerve ring surrounding anterior part of muscular oesophagus. Lateral alae well developed (Fig. 1e–f) tapering gradually to posterior end. Phasmids small, subterminal. Vulva simple, near tail end (Fig. 1g). Reproductive system monodelphic. Tail conical, with rounded tip (Fig. 1g). Eggs oval, embryonated, covered with small protuberances (Fig. 1h).

#### *Cosmocephalus obvelatus* (Creplin, 1825) Seurat, 1919

**Figure 2:**
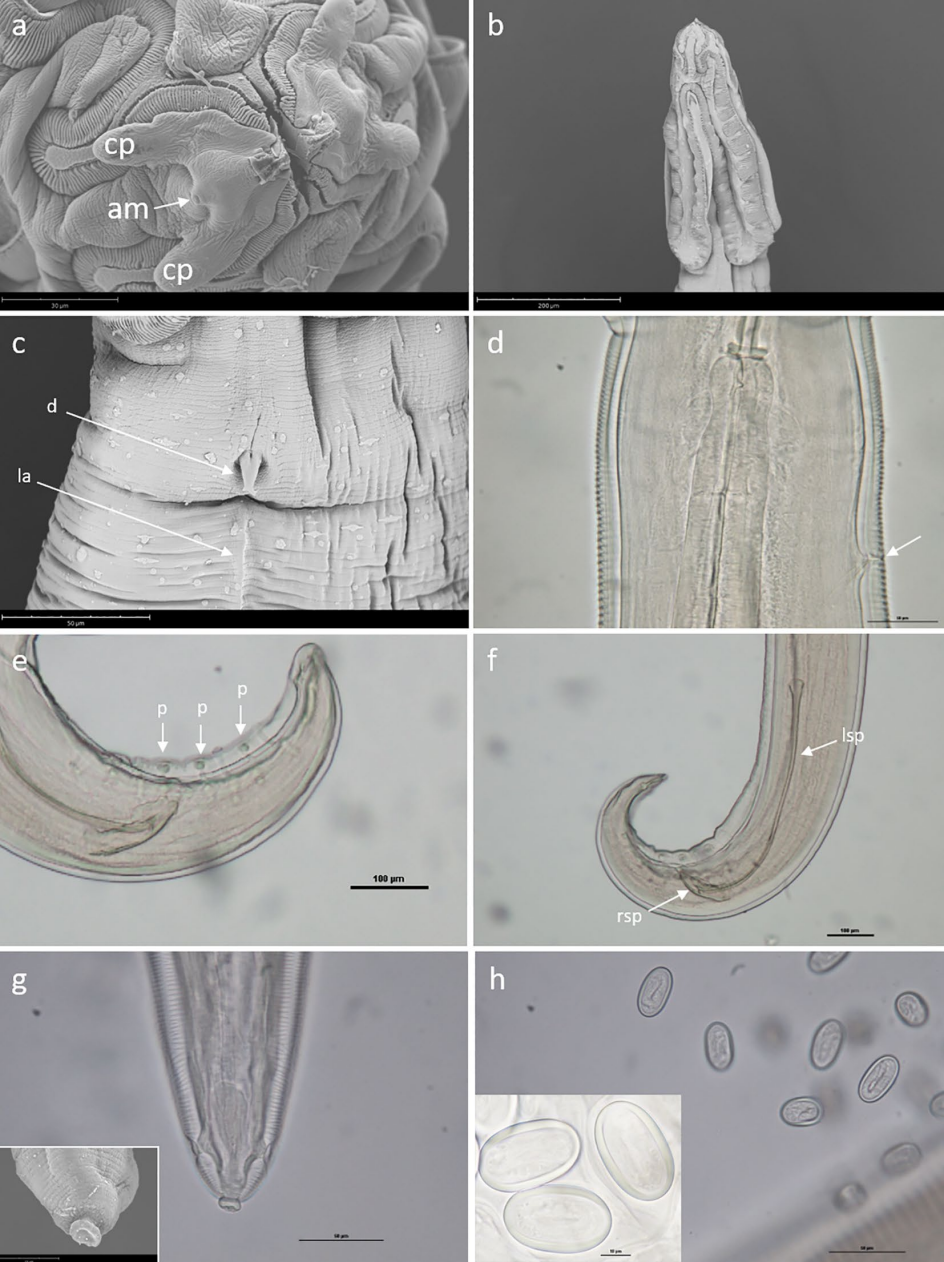
*Cosmocephalus obvelatus* from *Microcarbo pygmaeus*, light microscopy and SEM micrographs. (**a**) Apical view of anterior end, cephalic papillae (cp) and amphid (am) (scale bar = 30 µm). (**b**) Lateral view of anterior end showing the appearance of cordons (scale bar = 200 µm). (**c**) Bicuspid deirid (**d**) and detail of lateral ala (la) (scale bar = 50 µm). (**d**) Anterior portion of muscular oesophagus with nerve ring and excretory pore (arrow) (scale bar = 10 µm). (**e**) Posterior end of male showing caudal papillae (p) (scale bar = 100 µm). (**f**) Posterior end of male showing the appearance of left (lsp) and right (rsp) spicules (scale bar = 100 µm). (**g**) Posterior end of female showing characteristic button-like tip (scale bar = 50 µm) (inset: SEM micrograph. Scale bar = 15 µm). (**h**) Eggs (scale bar = 50 µm) (inset: detail of eggs at higher magnification. Scale bar = 10 µm).

Adult general morphology: oral opening slit-like, surrounded by two pseudolabia, each with a single amphid and a pair of papillae, and two bifid interlabia (Fig. 2a); cordons originating dorsally and ventrally to pseudolabia, anastomising laterally, with scalloped inner borders (Fig. 2b). Anterior loops of cordons do not exceed one quarter of the length of cordons. Deirids bicuspid (Fig. 2c). Lateral alae well developed, extending from level posterior to deirids (Fig. 2c) to around middle of body. Excretory pore posterior to deirids (Fig. 2d). Nerve ring surrounding anterior part of muscular oesophagus, approximately at level of deirids. Phasmids subterminal.

Adult male: Tail with ventral cuticular ridge, anterior to cloaca; ten pairs of caudal papillae, 4 pre-cloacal and 5 post-cloacal, and a single precloacal papilla, slightly anterior to cloacal opening (Fig. 2e). Spicules unequal: left spicule longer and narrower, with small projection on its distal end; right spicule shorter, and wider (Fig. 2f).

Adult female: Vulva around middle of body; lateral alae extending to level of vulva; uterus didelphic; tail conical, with characteristic button-like tip (Fig. 2g). Eggs oval, embryonated (Fig. 2h).

### Molecular analyses

All 13 specimens from Italy were successfully amplified. A BLAST search revealed that 12 samples exhibited 98.6% similarity with *Syncuaria sagittata* (Rudolphi, 1809) (MT086856) and *Decorataria decorata* (Cram, 1927) (MT086842^3^), while one specimen showed 99.4% similarity (33% coverage) with *Cosmocephalus jaenschi* Johnston and Mawson, 1941 (OP455796^16^).

**Figure 3:**
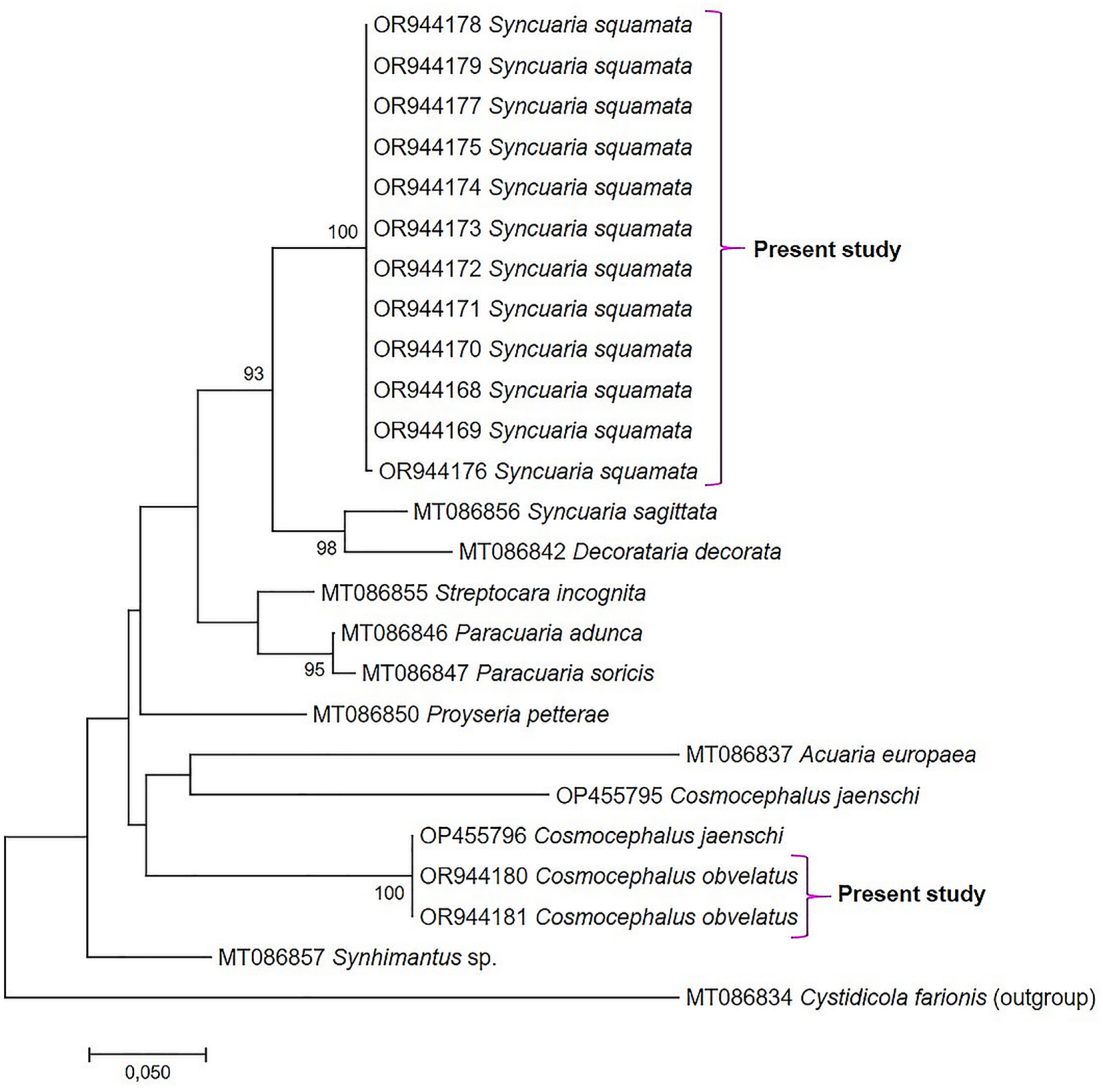
Maximum Likelihood (GTR model) tree based on 28S rDNA sequences newly generated together with other acuariids retrieved from GenBank. The tree is drawn to scale, with branch lengths measured in the number of substitutions per site. The analysis involved 25 nucleotide sequences.

Among the 7 specimens collected in Israel, only one gave a readable pherograms, probably due to preservation issues; the similarity was 99.4% with *C. jaenschi* (OP455796^16^). The p-distance analysis revealed that all the *Syncuaria* sequences obtained in this study were identical to each other and showed a 0.8% distance from *S. sagittata*. This confirms that they do not belong to the latter species, as observed through morphological identification, but rather to *S. squamata*, for which no sequences are available in GenBank. In fact, in the Maximum Likelihood (ML) tree (Fig. 3), our sequences form a well-supported cluster, distinctly separated from the cluster (98% bootstrap) formed by *S. sagittata* and *D. decorata*.

Concerning the *Cosmocephalus* sp., the two specimens (one from Italy and one from Israel) were identical to each other with a p-distance of 1.6% with *C. jaenschi* (OP455795^16^) but 0% with OP455796 (*C. jaenschi*^16^). These results coupled with the morphological observation allowed to state that our species was not *C. jaenschi* but *C. obvelatus* (no sequences available in GB) and move the specimen from sprat of Bennett et al.^16^ in the latter species; the ML tree confirmed this observation (100% bootstrap) (Fig. 3).

## Discussion

The genus *Syncuaria* Gilbert, 1927 comprises eight species parasitizing members of the Ciconiiformes and one species specific of grebes^17^. The genera *Skrjabinocara* Kurashvili, 1940 and *Decorataria* Sobolev, 1949 have been considered as synonyms of *Syncuaria*^13,18,19^, although other authors have retained the validity of the three genera^2,3,20^.

The species *S. squamata* has been reported from the proventriculus and gizzard of different cormorant species (*Ph. Carbo* (Linnaeus), *Ph. auritus* (Lesson, 1831), *Ph. brasilianus* (Gmelin, 1789) and *M. pygmaeus*) and from Accipitriformes^1,21^. It was originally described as *Filaria squamata* by Linstow^22^, then redescribed as *Skrjabinocara squamata* by Kurochkin^10^, and finally assigned to the genus *Syncuaria* by Wong et al.^13^. This species is widely distributed and reported from different areas of Europe, Asia, and America^1,23^.

In the definitive host, prevalence and intensity values can be highly variable: Kanarek and Rolbiecki^24^ found that *S. squamata* infection was heavier in the immature birds (84.6%, 40.9 worms, and 1–120 worms) than in the adults (3.7%, 1 worm). These differences may suggest either a possible acquired resistance or an ontogenetic dietary shift of the cormorants. Moravec and Scholz^23^ found prevalence values of 15% and 20% and intensity values of 1–12 (mean = 4) and 1–4 (mean = 2) in *Ph. carbo sinensis* from South Bohemia and South Moravia, respectively. In Italy, *S. squamata* has already been reported from great cormorants with a prevalence around 20% and intensity values ranging from 1 to 10^25^. In *Ph. carbo sinensis* examined during the present investigation, intensity of *S. squamata* was 14. This overall low intensity may be related to possible mechanisms of competitive interactions with other species of parasitic helminths, as suggested by Dezfuli et al.^25^; in fact, in the same *Ph. carbo sinensis* analyzed in the present work, high intensity values of the anisakid nematodes *Contracaecum rudolphii* A and *Contracaecum rudolphii* B were also observed^26^; the same *M. pygmaeus* analyzed in the present work, were infected also by *Amirthalingamia macracantha* (Joyeux and Baer 1935) (Cestoda)^27^.

Interestingly, in our study only female specimens were recovered. This finding is in accordance with the observation of Monteiro et al.^21^, who found only one male out of 9 *S. squamata* recovered from *Ph. brasilianus*. Morphological features, as observed in light and scanning electron microscopy, and morphometry (Table 1) are in accordance with previous studies^1,13,21,23,24,28^, although variability in the shape of deirids (both bifid and trifid) has been reported by some of these authors^21,23^. The only *Syncuaria* available in GenBank is the species *S. sagittata,* which in the ML tree is clearly separated from *S. squamata* described in the present study; the two species also show different morphological features. Particularly, *S. sagittata* was previously known as *Desportesius sagittatus* (Rudolphi, 1809)^13,29^ and was subsequently assigned to the genus *Syncuaria* based on phylogenetic analysis^3^ (Mutafchiev et al. 2020); this species is found in the gizzard of different birds (*Ardea purpurea* Linnaeus, 1766, *Ciconia nigra* Linnaeus, 1758, *Nycticorax nycticorax* Linnaeus, 1758)^1^ and is clearly distinguishable from *S. squamata* by its overall smaller size and by the morphology of anterior end, characterized by recurrent cordons reaching approximately half of the muscular oesophagus^30^. Therefore, our molecular data further support the separation between these two congeneric species.

The genus *Cosmocephalus* includes seven species^14^, all parasitic in aquatic birds. Particularly, the species *C. obvelatus* shows a cosmopolitan distribution and low specificity, being reported from a wide range of bird species^1,7^, including several species of cormorants (Phalacrocoracidae). In other groups of bird parasites (e.g. in Anisakidae of the genus *Contracaecum* Railliet & Henry, 1912), the use of molecular data has helped to reveal hidden genetic diversity^26^; however, as mentioned earlier, few sequence data of acuariids are available in public databases: for the genus *Cosmocephalus*, such data are available only for the species *C. jaenschi*. Nevertheless, the wide distribution of this species has also been associated to its ecology and to the wide-ranging ability of its hosts^7,31^.

*Cosmocephalus obvelatus* was previously reported in *Ph. carbo sinensis* from the European continent^32,33^ and in *M. pygmaeus*^1^; our study reports for the first time the occurrence of this nematode species in birds from Italy and Israel. Unidentified *Cosmocephalus* sp. was also reported in *M. pygmaeus* from Indonesia^34^ however morphological details provided are insufficient to confirm its specific identity. Another congeneric species reported from *M. pygmaeus* is *C. jaenschi*, which differed from *C. obvelatus* mainly for having tricuspid deirids (but bicuspid deirids have also been occasionally described, see Presswell and Bennett^35^ and different spicule morphometry. In particular, the size range of spicules from our specimens overlaps with that reported in recent detailed redescription of *C. obvelatus*^14,31^ and in older descriptions from Europe^1,36^, while it differs from the size range reported for *C. jaenschi*^14,35^. Both species are described as having an appendage on the distal end of the left spicule; however, in *C. jaenschi* this appendage appears more prominent than in *C. obvelatus*^14^. Furthermore, in our male specimens, the proximal pair of precloacal papillae lies slightly external to the distribution of the other precloacal papillae, a feature already reported for *C. obvelatus*^31^.

Interestingly, *C. jaenschi* has been reported mostly, although not exclusively, from the southern hemisphere^16,35,37–39^. Indeed, in the current study, the phylogenetic analysis revealed that the larval specimen identified as *C. jaenschi* (OP455796) by Bennet et al.^16^ in *Sprattus antipodum* (Hector, 1872) from New Zealand actually belongs to the species *C. obvelatus*. This conclusion is supported by a p-distance of 0% and a bootstrap value of 100%.

At present, morphological information on larval stages of most acuariid species is insufficient to allow their identification to the species level, further supporting the possibility of a misidentification.

The morphology of male and female *C. obvelatus* from our study are in accordance with other light and scanning electron microscopy studies of specimens from different hosts^7,14,31,40–42^.

This paper provides new data on the distribution of acuariid parasites of piscivorous birds, particularly in areas with no published information such as Israel. In addition, we provide the first genetic data of the species *S. squamata* and *C. obvelatus*, of possible usefulness for future epidemiological and phylogenetic studies on these widely distributed parasites.

## Data Availability

The DNA sequences generated in this study have been deposited on the public database GenBank under Accession Numbers OR944168-79 (*Syncuaria squamata*) and OR944180-81 (*Cosmocephalus obvelatus*).
